# 4-[(1-Hy­droxy-2-naphth­yl)methyl­ene­amino]-1,5-dimethyl-2-phenyl-1*H*-pyrazol-3(2*H*)-one

**DOI:** 10.1107/S1600536810026450

**Published:** 2010-07-10

**Authors:** Qiang Liang, Qian Wang

**Affiliations:** a Department of Chemistry, Teachers College of Qingdao University, Qingdao, Shandong 266071, People’s Republic of China

## Abstract

The title anti­pyrine derivative, C_22_H_19_N_3_O_2_, was synthesized by the reaction of 4-amino-1,5-dimethyl-2-phenyl-1,2-dihydro­pyrazol-3-one and 1-hy­droxy­naphthalene-2-carbaldehyde in methanol solution. As expected, the compound adopts a *trans* configuration about the central C=N bond. The N atom is involved in an intra­molecular O—H⋯N bond which stabilizes the mol­ecular configuration. In the crystal structure, adjacent mol­ecules stack with no short contacts.

## Related literature

For background to the applications of anti­pyrine derivatives, see: Bashkatova *et al.* (2005 ▶); Bansal *et al.* (2007 ▶); Bondock *et al.* (2008 ▶); Capel *et al.* (1978 ▶); Coolen *et al.* (1999 ▶); Collado *et al.* (2000 ▶); Cunha *et al.* (2005 ▶); Evstropov *et al.* (1992 ▶); Khanduja *et al.* (1984 ▶); Madiha *et al.* (2007 ▶); Plesch *et al.* (1987 ▶); Radzikowska *et al.* (1995 ▶); Rehim *et al.* (2001 ▶); Turan-Zitouni *et al.* (2001 ▶); Yadav *et al.* (2003 ▶). For some typical structures of anti­pyrine derivatives, see: Liang *et al.* (2002 ▶); Li & Zhang (2004 ▶, 2005 ▶); Sun, Xie *et al.* (2006 ▶); Sun, Zhang, Jin *et al.* (2006 ▶); Sun, Zhang, Wang *et al.* (2006 ▶); Sun, Hao, Wei *et al.* (2009 ▶); Wen *et al.* (2005 ▶); You *et al.* (2004 ▶, 2006 ▶); Zhang & Li *et al.* (2005 ▶). For related structures involving Schiff bases, see: Ali *et al.* (2002 ▶); Bashkatova *et al.* (2005 ▶); Coolen *et al.* (1999 ▶); Collado *et al.* (2000 ▶); Cukurovali *et al.* (2002 ▶); Farag *et al.* (2009 ▶); Rehim *et al.* (2001 ▶); Sun, Hao, Yu *et al.* (2009 ▶); Tarafder *et al.* (2002 ▶).
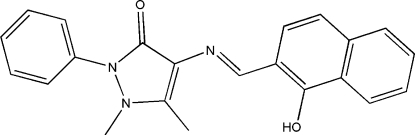


## Experimental

### 

#### Crystal data

C_22_H_19_N_3_O_2_
*M* *_r_* = 357.40
Monoclinic, 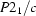

*a* = 8.0636 (7) Å
*b* = 7.4407 (6) Å
*c* = 30.169 (3) Å
β = 94.329 (2)°
*V* = 1804.9 (3) Å^3^
*Z* = 4
Mo *K*α radiation
μ = 0.09 mm^−1^
*T* = 295 K
0.23 × 0.10 × 0.02 mm

#### Data collection

Bruker APEX area-detector diffractometer
Absorption correction: multi-scan (*SADABS*; Sheldrick, 1996 ▶) *T*_min_ = 0.981, *T*_max_ = 0.998
14746 measured reflections
3942 independent reflections
2403 reflections with *I* > 2σ(*I*)
*R*_int_ = 0.050

#### Refinement

*R*[*F* ^2^ > 2σ(*F* ^2^)] = 0.060
*wR*(*F* ^2^) = 0.147
*S* = 1.04
3942 reflections
252 parameters
H-atom parameters constrained
Δρ_max_ = 0.14 e Å^−3^
Δρ_min_ = −0.17 e Å^−3^

### 

Data collection: *SMART* (Bruker, 2002 ▶); cell refinement: *SAINT-Plus* (Bruker, 2002 ▶); data reduction: *SAINT-Plus*; program(s) used to solve structure: *SHELXS97* (Sheldrick, 2008 ▶); program(s) used to refine structure: *SHELXL97* (Sheldrick, 2008 ▶); molecular graphics: *SHELXTL* (Sheldrick, 2008 ▶); software used to prepare material for publication: *SHELXTL*.

## Supplementary Material

Crystal structure: contains datablocks global, I. DOI: 10.1107/S1600536810026450/gw2081sup1.cif

Structure factors: contains datablocks I. DOI: 10.1107/S1600536810026450/gw2081Isup2.hkl

Additional supplementary materials:  crystallographic information; 3D view; checkCIF report

## Figures and Tables

**Table 1:** Hydrogen-bond geometry (Å, °)

*D*—H⋯*A*	*D*—H	H⋯*A*	*D*⋯*A*	*D*—H⋯*A*
O2—H2⋯N3	0.82	1.84	2.569 (2)	148

**Fig. 1.**
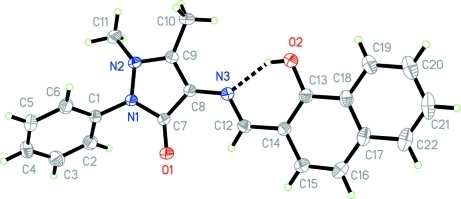
The molecular structure of (I), showing 30% probability displacement ellipsoids and the atom-numbering scheme. The O—H···O hydrogen bond is shown as a dashed line.

**Fig. 2.**
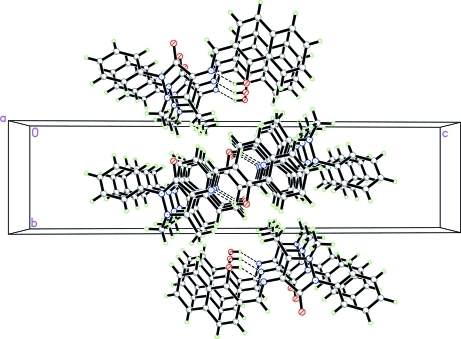
The crystal packing of (I), viewed down the a axis. O—H···N contacts are shown as Hydrogen bonds.

## supplementary crystallographic information

### Comment

Since antipyrine was first synthesized by Knorr in 1883, the antipyrine and its derivatives exhibit a wide range of biologcial or chemical activities and applications (Capel *et al.*, 1978; Radzikowska *et al.*, 1995; Khanduja *et al.*, 1984; Bondock *et al.*, 2008; Cunha *et al.*, 2005; Plesch *et al.*, 1987; Madiha *et al.*, 2007; Evstropov *et al.*, 1992; Turan-Zitouni *et al.*, 2001; Bansal *et al.*, 2007; Bashkatova *et al.*, 2005; Rehim *et al.*, 2001; Collado *et al.*, 2000; Coolen *et al.*, 1999; Yadav *et al.*, 2003). A few crystal structures of antipyrine derivatives have been investigated (Liang *et al.*, 2002; Li & Zhang, 2004, 2005; Zhang & Li, 2005; You, *et al.*, 2004, 2006; Wen, 2005; Sun, Zhang, Jin *et al.*, 2006, Sun, Zhang, Wang *et al.*, 2006; Sun, Xie  *et al.*, 2006 ; Sun, Hao, Wei *et al.* 2009). Schiff bases condensed by aldehydes and amines have demonstrated significant biological, chemical or optical activites, and new examples are being tested for their antitumor, antimicrobial, antiviral, antioxidant, optical and photovoltaic activities (Tarafder *et al.*, 2002; Cukurovali *et al.*, 2002; Ali *et al.*, 2002; Bashkatova *et al.*, 2005; Rehim *et al.*, 2001; Collado *et al.*, 2000; Coolen *et al.*, 1999; Sun, Hao, Yu *et al.*, 2009; Farag *et al.*, 2009). As an extension of these works on the structural characterization of antipyrine derivatives, a new Schiff base compound, (I), is reported here.

As illustrated in Fig. 1, the compound (I) is a neutral 4-((1-hydroxynaphthalen-2-yl)methyleneamino)-1,2-dihydro-1,5-dimethyl- 2-phenylpyrazol-3-one molecule. Selected geometric parameters are listed in Table 1. The N2-N1-C1-C2 and C7-N1-C1-C6 torsion angles are 147.2 (2) and 115.1 (2) °, respectively. Atom O1 deviates from the pyrazoline mean plane by 0.140 (2) Å, whereas atom C10 and C11 deviate from it, on the opposite side, by 0.087 (2) and 0.614 (2) Å, respectively. The dihedral angle between the N1/N2/C7/C8/C9 pyrazoline ring and the C1-C6 benzene ring planes is 50.4 (3) °. The C12═N3 bond length of 1.290 (3) Å confirms to the value for a double bond. As a result of conjugation through the imino double bond, the C12-N3-C8-C9 and C12-N3-C8-C7 torsion angles are 172.8 (2) and -2.5 (3) ° respectively, the pyrazoline and C13-C22 naphthalene rings are nearly coplannar [mean deviation from the overall combined mean plane is 0.084 (3) Å]; the dihedral angle between the pyrazoline ring and C13-C22 naphthalene ring is 11.5 (3) °. As expected, the molecular structure of the Shiff base adopts a *trans* coonfigurations about the central C12═N3 bond as the other similar antipyrine derivatives that have been reported.

In the crystal structure, the molecules stack along the *a* axis with no short contacts except the O—H···N intramolecular hydrogen (Table 2 and Fig. 2).

### Experimental

All the chemicals were obtained from commercial sources and used without purification. 4-amino-1,5-dimethyl-2-phenyl-1,2-dihydropyrazole-3-one (0.5 mmol, 101.6 mg) and an equimolar quantity of 1-hydroxynaphthalene- 2-carbaldehyde (0.5 mmol, 86.1 mg) were dissolved in methanol (100 ml). The mixture was stirred for 1 h at room temperature to give a clear yellow solution. The resulting solution was kept in air for 8 d after which time yellow plane-shaped crystals of (I) were formed at the bottom of the vessel on slow evaporation of the methanol. (yield 95.2%). Analysis calculated for (C_18_H_16_ClN_3_O_2_): C 73.93, H 5.36, N 11.76%; found: C 73.55, H 5.42, N 11.73%.

### Refinement

All H atoms were positioned geometrically (O—H = 0.82 Å and C—H = 0.93 or 0.96 Å) and constrained to ride on their parent atoms with *U*_iso_(H) = 1.5*U*_eq_(O) for phenolic H atom, *U*_iso_(H) = 1.2 for aromatic H atoms or *U*_iso_(H) = 1.5*U*_eq_(C) for methyl H atoms.

### Crystal data

**Table:**

C_22_H_19_N_3_O_2_	*F*(000) = 752
*M**_r_* = 357.40	*D*_x_ = 1.315 Mg m^−^^3^
Monoclinic, *P*2_1_/*c*	Mo *K*α radiation, λ = 0.71073 Å
Hall symbol: -P 2ybc	Cell parameters from 1592 reflections
*a* = 8.0636 (7) Å	θ = 2.5–25.1°
*b* = 7.4407 (6) Å	µ = 0.09 mm^−^^1^
*c* = 30.169 (3) Å	*T* = 295 K
β = 94.329 (2)°	Plane, yellow
*V* = 1804.9 (3) Å^3^	0.23 × 0.10 × 0.02 mm
*Z* = 4	

### Data collection

**Table:**

Bruker APEX area-detector diffractometer	3942 independent reflections
Radiation source: fine-focus sealed tube	2403 reflections with *I* > 2σ(*I*)
graphite	*R*_int_ = 0.050
φ and ω scans	θ_max_ = 27.0°, θ_min_ = 1.4°
Absorption correction: multi-scan (*SADABS*; Sheldrick, 1996)	*h* = −10→10
*T*_min_ = 0.981, *T*_max_ = 0.998	*k* = −9→9
14746 measured reflections	*l* = −37→38

### Refinement

**Table:**

Refinement on *F*^2^	Primary atom site location: structure-invariant direct methods
Least-squares matrix: full	Secondary atom site location: difference Fourier map
*R*[*F*^2^ > 2σ(*F*^2^)] = 0.060	Hydrogen site location: inferred from neighbouring sites
*wR*(*F*^2^) = 0.147	H-atom parameters constrained
*S* = 1.04	*w* = 1/[σ^2^(*F*_o_^2^) + (0.0584*P*)^2^ + 0.2473*P*] where *P* = (*F*_o_^2^ + 2*F*_c_^2^)/3
3942 reflections	(Δ/σ)_max_ < 0.001
252 parameters	Δρ_max_ = 0.14 e Å^−^^3^
0 restraints	Δρ_min_ = −0.17 e Å^−^^3^

### Special details

**Table:**

Geometry. All esds (except the esd in the dihedral angle between two l.s. planes) are estimated using the full covariance matrix. The cell esds are taken into account individually in the estimation of esds in distances, angles and torsion angles; correlations between esds in cell parameters are only used when they are defined by crystal symmetry. An approximate (isotropic) treatment of cell esds is used for estimating esds involving l.s. planes.
Refinement. Refinement of F^2^ against ALL reflections. The weighted R-factor wR and goodness of fit S are based on F^2^, conventional R-factors R are based on F, with F set to zero for negative F^2^. The threshold expression of F^2^ > 2sigma(F^2^) is used only for calculating R-factors(gt) etc. and is not relevant to the choice of reflections for refinement. R-factors based on F^2^ are statistically about twice as large as those based on F, and R- factors based on ALL data will be even larger.

### Fractional atomic coordinates and isotropic or equivalent isotropic displacement parameters (Å^2^)

**Table:**

	*x*	*y*	*z*	*U*_iso_*/*U*_eq_	Occ. (<1)
O1	0.5537 (2)	0.63818 (19)	0.63468 (5)	0.0518 (4)	
O2	0.2652 (2)	0.2809 (2)	0.48208 (5)	0.0529 (5)	
H2	0.3246	0.2763	0.5053	0.079*	
N1	0.6496 (2)	0.3617 (2)	0.66126 (6)	0.0430 (5)	
N2	0.6208 (2)	0.1816 (2)	0.64902 (6)	0.0440 (5)	
N3	0.4118 (2)	0.3947 (2)	0.55528 (6)	0.0391 (4)	
C1	0.6836 (3)	0.4067 (3)	0.70681 (7)	0.0416 (5)	
C2	0.7888 (3)	0.5480 (3)	0.71763 (8)	0.0546 (6)	
H2A	0.8380	0.6109	0.6954	0.066*	
C3	0.8210 (3)	0.5961 (4)	0.76159 (9)	0.0662 (8)	
H3	0.8910	0.6926	0.7690	0.079*	
C4	0.7503 (4)	0.5023 (4)	0.79433 (9)	0.0699 (8)	
H4	0.7726	0.5351	0.8239	0.084*	
C5	0.6471 (4)	0.3607 (4)	0.78370 (8)	0.0633 (7)	
H5	0.6007	0.2963	0.8061	0.076*	
C6	0.6115 (3)	0.3130 (3)	0.73982 (8)	0.0526 (6)	
H6	0.5393	0.2182	0.7325	0.063*	
C7	0.5673 (3)	0.4746 (3)	0.62969 (7)	0.0383 (5)	
C8	0.5054 (3)	0.3554 (3)	0.59459 (7)	0.0366 (5)	
C9	0.5442 (3)	0.1841 (3)	0.60707 (7)	0.0408 (5)	
C10	0.513 (2)	0.016 (3)	0.5821 (7)	0.0599 (11)	0.68 (4)
H10A	0.4444	0.0406	0.5554	0.090*	0.68 (4)
H10B	0.4572	−0.0679	0.6001	0.090*	0.68 (4)
H10C	0.6168	−0.0343	0.5746	0.090*	0.68 (4)
C10'	0.501 (5)	0.018 (7)	0.5804 (16)	0.0599 (11)	0.32 (4)
H10D	0.3824	0.0105	0.5746	0.090*	0.32 (4)
H10E	0.5402	−0.0861	0.5969	0.090*	0.32 (4)
H10F	0.5525	0.0231	0.5528	0.090*	0.32 (4)
C11	0.7486 (3)	0.0508 (3)	0.66333 (8)	0.0575 (7)	
H11A	0.7126	−0.0673	0.6541	0.086*	
H11B	0.7672	0.0539	0.6951	0.086*	
H11C	0.8501	0.0797	0.6502	0.086*	
C12	0.3597 (3)	0.5544 (3)	0.54508 (7)	0.0412 (5)	
H12	0.3903	0.6499	0.5638	0.049*	
C13	0.2085 (3)	0.4488 (3)	0.47564 (7)	0.0404 (5)	
C14	0.2546 (3)	0.5875 (3)	0.50496 (7)	0.0382 (5)	
C15	0.1916 (3)	0.7620 (3)	0.49565 (8)	0.0496 (6)	
H15	0.2230	0.8555	0.5150	0.059*	
C16	0.0877 (3)	0.7968 (3)	0.45970 (8)	0.0562 (7)	
H16	0.0489	0.9132	0.4546	0.067*	
C17	0.0366 (3)	0.6577 (3)	0.42957 (7)	0.0497 (6)	
C18	0.0988 (3)	0.4819 (3)	0.43735 (7)	0.0444 (6)	
C19	0.0465 (3)	0.3427 (4)	0.40779 (8)	0.0570 (7)	
H19	0.0867	0.2266	0.4126	0.068*	
C20	−0.0626 (4)	0.3776 (5)	0.37217 (9)	0.0740 (9)	
H20	−0.0968	0.2849	0.3529	0.089*	
C21	−0.1237 (3)	0.5509 (5)	0.36419 (9)	0.0755 (9)	
H21	−0.1976	0.5730	0.3396	0.091*	
C22	−0.0758 (3)	0.6875 (4)	0.39216 (8)	0.0660 (8)	
H22	−0.1178	0.8024	0.3866	0.079*	

### Atomic displacement parameters (Å^2^)

**Table:**

	*U*^11^	*U*^22^	*U*^33^	*U*^12^	*U*^13^	*U*^23^
O1	0.0777 (12)	0.0296 (9)	0.0462 (10)	0.0008 (8)	−0.0081 (8)	−0.0028 (7)
O2	0.0680 (12)	0.0423 (10)	0.0465 (10)	0.0065 (8)	−0.0091 (8)	−0.0060 (8)
N1	0.0618 (12)	0.0285 (10)	0.0368 (10)	−0.0024 (9)	−0.0082 (9)	−0.0010 (8)
N2	0.0610 (13)	0.0258 (10)	0.0432 (11)	0.0017 (9)	−0.0086 (9)	0.0001 (8)
N3	0.0464 (11)	0.0342 (10)	0.0362 (10)	0.0010 (8)	0.0006 (8)	−0.0002 (8)
C1	0.0508 (14)	0.0365 (12)	0.0359 (12)	0.0005 (10)	−0.0071 (10)	0.0004 (10)
C2	0.0617 (16)	0.0486 (15)	0.0519 (15)	−0.0066 (12)	−0.0064 (13)	−0.0003 (12)
C3	0.0732 (19)	0.0597 (18)	0.0620 (18)	−0.0080 (15)	−0.0195 (15)	−0.0113 (15)
C4	0.095 (2)	0.071 (2)	0.0398 (15)	0.0118 (17)	−0.0171 (15)	−0.0065 (14)
C5	0.088 (2)	0.0609 (17)	0.0401 (15)	0.0049 (16)	0.0006 (14)	0.0079 (13)
C6	0.0630 (16)	0.0472 (15)	0.0461 (15)	−0.0066 (12)	−0.0055 (12)	0.0051 (11)
C7	0.0456 (13)	0.0316 (12)	0.0372 (12)	−0.0003 (10)	−0.0001 (10)	0.0023 (9)
C8	0.0416 (12)	0.0305 (11)	0.0372 (12)	−0.0015 (10)	−0.0014 (10)	−0.0018 (9)
C9	0.0476 (13)	0.0349 (12)	0.0395 (13)	−0.0029 (10)	0.0003 (10)	−0.0028 (9)
C10	0.091 (3)	0.0295 (14)	0.057 (2)	0.000 (2)	−0.013 (3)	−0.0056 (15)
C10'	0.091 (3)	0.0295 (14)	0.057 (2)	0.000 (2)	−0.013 (3)	−0.0056 (15)
C11	0.0692 (17)	0.0410 (14)	0.0601 (16)	0.0112 (12)	−0.0110 (13)	0.0041 (12)
C12	0.0460 (13)	0.0374 (13)	0.0401 (13)	−0.0028 (10)	0.0019 (10)	−0.0029 (10)
C13	0.0434 (13)	0.0402 (13)	0.0379 (12)	−0.0006 (10)	0.0048 (10)	0.0023 (10)
C14	0.0410 (12)	0.0358 (12)	0.0377 (12)	−0.0015 (10)	0.0031 (10)	0.0029 (9)
C15	0.0563 (15)	0.0399 (13)	0.0519 (15)	0.0023 (11)	−0.0003 (12)	0.0023 (11)
C16	0.0575 (16)	0.0499 (15)	0.0604 (17)	0.0077 (13)	0.0001 (13)	0.0140 (13)
C17	0.0417 (14)	0.0658 (17)	0.0417 (14)	0.0009 (12)	0.0027 (11)	0.0149 (12)
C18	0.0413 (13)	0.0564 (15)	0.0357 (12)	−0.0070 (11)	0.0043 (10)	0.0045 (11)
C19	0.0570 (16)	0.0698 (18)	0.0435 (14)	−0.0126 (13)	−0.0011 (12)	−0.0044 (13)
C20	0.0669 (19)	0.105 (3)	0.0485 (17)	−0.0213 (18)	−0.0042 (14)	−0.0058 (17)
C21	0.0522 (17)	0.131 (3)	0.0420 (16)	−0.0102 (19)	−0.0091 (13)	0.0173 (18)
C22	0.0512 (16)	0.093 (2)	0.0533 (17)	0.0027 (15)	−0.0003 (13)	0.0269 (16)

### Geometric parameters (Å, °)

**Table:**

O1—C7	1.232 (2)	C10—H10C	0.9600
O2—C13	1.340 (2)	C10'—H10D	0.9600
O2—H2	0.8200	C10'—H10E	0.9600
N1—C7	1.399 (3)	C10'—H10F	0.9600
N1—N2	1.405 (2)	C11—H11A	0.9600
N1—C1	1.421 (3)	C11—H11B	0.9600
N2—C9	1.366 (3)	C11—H11C	0.9600
N2—C11	1.459 (3)	C12—C14	1.445 (3)
N3—C12	1.290 (3)	C12—H12	0.9300
N3—C8	1.388 (3)	C13—C14	1.391 (3)
C1—C2	1.375 (3)	C13—C18	1.422 (3)
C1—C6	1.380 (3)	C14—C15	1.415 (3)
C2—C3	1.379 (3)	C15—C16	1.345 (3)
C2—H2A	0.9300	C15—H15	0.9300
C3—C4	1.369 (4)	C16—C17	1.418 (3)
C3—H3	0.9300	C16—H16	0.9300
C4—C5	1.365 (4)	C17—C22	1.411 (3)
C4—H4	0.9300	C17—C18	1.414 (3)
C5—C6	1.380 (3)	C18—C19	1.410 (3)
C5—H5	0.9300	C19—C20	1.362 (3)
C6—H6	0.9300	C19—H19	0.9300
C7—C8	1.441 (3)	C20—C21	1.395 (4)
C8—C9	1.359 (3)	C20—H20	0.9300
C9—C10	1.47 (3)	C21—C22	1.358 (4)
C9—C10'	1.50 (6)	C21—H21	0.9300
C10—H10A	0.9600	C22—H22	0.9300
C10—H10B	0.9600		
			
C13—O2—H2	109.5	H10D—C10'—H10E	109.5
C7—N1—N2	109.49 (16)	C9—C10'—H10F	109.5
C7—N1—C1	124.31 (17)	H10D—C10'—H10F	109.5
N2—N1—C1	119.69 (16)	H10E—C10'—H10F	109.5
C9—N2—N1	106.54 (16)	N2—C11—H11A	109.5
C9—N2—C11	122.92 (18)	N2—C11—H11B	109.5
N1—N2—C11	117.36 (18)	H11A—C11—H11B	109.5
C12—N3—C8	123.00 (18)	N2—C11—H11C	109.5
C2—C1—C6	120.1 (2)	H11A—C11—H11C	109.5
C2—C1—N1	118.7 (2)	H11B—C11—H11C	109.5
C6—C1—N1	121.2 (2)	N3—C12—C14	121.1 (2)
C1—C2—C3	119.7 (2)	N3—C12—H12	119.4
C1—C2—H2A	120.2	C14—C12—H12	119.4
C3—C2—H2A	120.2	O2—C13—C14	121.88 (19)
C4—C3—C2	120.2 (3)	O2—C13—C18	117.6 (2)
C4—C3—H3	119.9	C14—C13—C18	120.5 (2)
C2—C3—H3	119.9	C13—C14—C15	118.7 (2)
C5—C4—C3	120.2 (2)	C13—C14—C12	121.15 (19)
C5—C4—H4	119.9	C15—C14—C12	120.1 (2)
C3—C4—H4	119.9	C16—C15—C14	122.0 (2)
C4—C5—C6	120.2 (3)	C16—C15—H15	119.0
C4—C5—H5	119.9	C14—C15—H15	119.0
C6—C5—H5	119.9	C15—C16—C17	120.7 (2)
C5—C6—C1	119.6 (2)	C15—C16—H16	119.7
C5—C6—H6	120.2	C17—C16—H16	119.7
C1—C6—H6	120.2	C22—C17—C18	118.5 (2)
O1—C7—N1	123.52 (19)	C22—C17—C16	122.4 (2)
O1—C7—C8	131.94 (19)	C18—C17—C16	119.1 (2)
N1—C7—C8	104.51 (17)	C19—C18—C17	119.3 (2)
C9—C8—N3	122.23 (19)	C19—C18—C13	121.6 (2)
C9—C8—C7	108.31 (19)	C17—C18—C13	119.1 (2)
N3—C8—C7	129.33 (18)	C20—C19—C18	120.2 (3)
C8—C9—N2	110.37 (18)	C20—C19—H19	119.9
C8—C9—C10	128.9 (9)	C18—C19—H19	119.9
N2—C9—C10	120.7 (9)	C19—C20—C21	120.8 (3)
C8—C9—C10'	125.8 (18)	C19—C20—H20	119.6
N2—C9—C10'	123.7 (18)	C21—C20—H20	119.6
C10—C9—C10'	4(2)	C22—C21—C20	120.3 (3)
C9—C10—H10A	109.5	C22—C21—H21	119.9
C9—C10—H10B	109.5	C20—C21—H21	119.9
C9—C10—H10C	109.5	C21—C22—C17	121.0 (3)
C9—C10'—H10D	109.5	C21—C22—H22	119.5
C9—C10'—H10E	109.5	C17—C22—H22	119.5
			
C7—N1—N2—C9	9.2 (2)	C11—N2—C9—C8	−147.1 (2)
C1—N1—N2—C9	162.14 (19)	N1—N2—C9—C10	173.1 (9)
C7—N1—N2—C11	151.62 (19)	C11—N2—C9—C10	33.4 (9)
C1—N1—N2—C11	−55.5 (3)	N1—N2—C9—C10'	176.1 (18)
C7—N1—C1—C2	−64.1 (3)	C11—N2—C9—C10'	36.4 (18)
N2—N1—C1—C2	147.2 (2)	C8—N3—C12—C14	−176.44 (19)
C7—N1—C1—C6	115.0 (2)	O2—C13—C14—C15	179.1 (2)
N2—N1—C1—C6	−33.6 (3)	C18—C13—C14—C15	−0.7 (3)
C6—C1—C2—C3	−0.4 (4)	O2—C13—C14—C12	−3.1 (3)
N1—C1—C2—C3	178.8 (2)	C18—C13—C14—C12	177.09 (19)
C1—C2—C3—C4	0.8 (4)	N3—C12—C14—C13	−1.2 (3)
C2—C3—C4—C5	−0.1 (4)	N3—C12—C14—C15	176.6 (2)
C3—C4—C5—C6	−1.0 (4)	C13—C14—C15—C16	0.7 (3)
C4—C5—C6—C1	1.4 (4)	C12—C14—C15—C16	−177.1 (2)
C2—C1—C6—C5	−0.7 (4)	C14—C15—C16—C17	0.2 (4)
N1—C1—C6—C5	−179.9 (2)	C15—C16—C17—C22	178.2 (2)
N2—N1—C7—O1	170.7 (2)	C15—C16—C17—C18	−1.1 (3)
C1—N1—C7—O1	19.4 (3)	C22—C17—C18—C19	−0.2 (3)
N2—N1—C7—C8	−7.4 (2)	C16—C17—C18—C19	179.1 (2)
C1—N1—C7—C8	−158.8 (2)	C22—C17—C18—C13	−178.2 (2)
C12—N3—C8—C9	172.8 (2)	C16—C17—C18—C13	1.1 (3)
C12—N3—C8—C7	−2.5 (3)	O2—C13—C18—C19	2.0 (3)
O1—C7—C8—C9	−175.0 (2)	C14—C13—C18—C19	−178.2 (2)
N1—C7—C8—C9	2.9 (2)	O2—C13—C18—C17	179.96 (19)
O1—C7—C8—N3	0.8 (4)	C14—C13—C18—C17	−0.2 (3)
N1—C7—C8—N3	178.7 (2)	C17—C18—C19—C20	0.0 (3)
N3—C8—C9—N2	−173.34 (19)	C13—C18—C19—C20	178.0 (2)
C7—C8—C9—N2	2.8 (3)	C18—C19—C20—C21	0.3 (4)
N3—C8—C9—C10	6.1 (10)	C19—C20—C21—C22	−0.5 (4)
C7—C8—C9—C10	−177.7 (9)	C20—C21—C22—C17	0.3 (4)
N3—C8—C9—C10'	3.1 (18)	C18—C17—C22—C21	0.1 (4)
C7—C8—C9—C10'	179.3 (18)	C16—C17—C22—C21	−179.2 (2)
N1—N2—C9—C8	−7.4 (3)		

### Hydrogen-bond geometry (Å, °)

**Table:**

*D*—H···*A*	*D*—H	H···*A*	*D*···*A*	*D*—H···*A*
O2—H2···N3	0.82	1.84	2.569 (2)	148
